# Outcomes of patients with relapsed/refractory acute leukaemia treated with revumenib with a focus on post‐revumenib therapies

**DOI:** 10.1111/bjh.70225

**Published:** 2025-10-26

**Authors:** Miles Thomas, Hannah Johnston, Emily Dworkin, Austin Wesevich, Gregory W. Roloff, Caner Saygin, Mariam T. Nawas, Michael W. Drazer, Adam S. DuVall, Satyajit Kosuri, Michael J. Thirman, Olatoyosi Odenike, Wendy Stock, Richard A. Larson, Rafael Madero‐Marroquin, Anand A. Patel

**Affiliations:** ^1^ Department of Medicine University of Chicago Chicago Illinois USA; ^2^ Section of Hematology and Oncology, Department of Medicine University of Chicago Chicago Illinois USA; ^3^ Department of Pharmacy University of Chicago Chicago Illinois USA; ^4^ University of Chicago Comprehensive Cancer Center Chicago Illinois USA

**Keywords:** acute lymphoblastic leukaemia, acute myeloid leukaemia, Revumenib

To the Editor,

Rearrangements of the lysine methyltransferase 2A gene (*KMT2A*r) and mutations in nucleophosmin 1 (*NPM1*m) are among the most common genetic aberrations in acute leukaemia, with *KMT2A*r seen in both acute myeloid leukaemia (AML) and acute lymphoblastic leukaemia (ALL) and *NPM1*m present in ~30% of AML cases.^1^ Historically, outcomes in relapsed/refractory (R/R) acute leukaemia with *KMT2A*r or *NPM1*m have been poor, with a median overall survival of 6 months or less.^2^, ^3^ Both *KMT2A*r and *NPM1*m acute leukaemia harbour leukaemogenic pathways dependent on aberrant transcription and differentiation blocks due to the protein menin. Revumenib, an oral selective menin inhibitor, disrupts leukaemogenesis in both *KMT2A*r and *NPM1*m acute leukaemias.^4^ The phase 1/2 AUGMENT‐101 trial of revumenib in patients with R/R *KMT2A*r and *NPM1*m acute leukaemia demonstrated a complete remission (CR) or complete remission with partial haematological recovery (CRh) rate of 22.8% among patients with *KMT2A*r and a CR + CRh rate of 23.4% in those with *NPM1*m.^5^, ^6^ While the single‐agent efficacy of revumenib in the R/R setting is promising, the duration of response has been limited; the median overall survival (OS) was 8 months for patients with R/R AML with *KMT2A*r and 4 months for those with *NPM1*m AML.^5^, ^6^ Currently, there are limited data regarding outcomes of subsequent therapies in patients already treated with revumenib. We sought to analyse the outcomes of patients with R/R acute leukaemia after receiving revumenib and the efficacy of subsequent treatment lines.

Adult patients with R/R acute leukaemia treated with revumenib monotherapy at the University of Chicago between 22 January 2020 and 22 May 2025 were studied as part of a single‐centre, retrospective cohort analysis. Patients were identified through the University of Chicago leukaemia registry and pharmacy records. Institutional review board approval was obtained. Diagnosis, relapse and disease status were confirmed according to the International Consensus Classification of myeloid neoplasms and acute leukaemias.^7^ Risk classification and response assessment for patients with AML utilized the European LeukemiaNet (ELN) 2022 criteria for intensive chemotherapy.^8^ Response assessments for patients with ALL and mixed‐phenotype acute leukaemia (MPAL) utilized the ELN 2024 adult ALL criteria.^9^

Descriptive statistics were utilized for baseline patient characteristics. A response was defined as achieving a CR, CRh or CR with incomplete count recovery (CRi); overall response rate (ORR) was defined as CR + CRh + CRi. OS was estimated using the Kaplan–Meier method.

We evaluated 26 patients treated with revumenib monotherapy for R/R acute leukaemia in our analysis. Twenty patients (76.9%) received revumenib as part of a clinical trial, 4 (15.4%) as part of an expanded access protocol and 2 (7.7%) as a commercial drug. Most patients (73%) had AML; the remainder had B‐ALL (15%) or rare subtypes (12%). Demographic and biological characteristics are presented in Table 1.

**TABLE 1:** Demographic and clinical characteristics of patients treated with revumenib and for the subset who received post‐revumenib therapy.

	All patients, *N* = 26 (%)	Patients who received post‐revumenib therapy (*n* = 15)
Median age at revumenib initiation in years [range]	58 [20, 85]	47 [20, 77]
Male sex	11 (42%)	6 (40%)
Race & ethnicity		
Non‐Hispanic White	18 (69%)	12 (80%)
Non‐Hispanic Black	4 (15%)	1 (7%)
Non‐Hispanic Asian	1 (4%)	0 (0%)
Hispanic	3 (12%)	2 (13%)
Leukaemia subtype		
AML	19 (73%)	11 (73%)
MDS/AML	1 (4%)	1 (7%)
MPAL	1 (4%)	0 (0%)
B‐ALL	4 (15%)	3 (20%)
T‐ALL	1 (4%)	0 (0%)
ELN 2022 risk classification at diagnosis^a^		
Favourable	4 (21%)	3 (27%)
Intermediate	7 (37%)	4 (37%)
Adverse	8 (42%)	4 (37%)
Indication for revumenib		
*KMT2A* rearranged	15 (58%)	9 (60%)
*KMT2A* partial tandem duplication	1 (4%)	1 (7%)
*NPM1* mutated	10 (38%)	5 (33%)
Lines of therapy pre‐revumenib		
≤2	16 (62%)	10 (67%)
≥3	10 (38%)	5 (33%)
Allo‐HSCT pre‐revumenib	7 (27%)	5 (33%)
Median duration of revumenib treatment in days [interquartile range]	59 [84]	65 [60]
Response to revumenib treatment		
CR	3 (12%)	2 (13%)
Cri	7 (27%)	3 (20%)
CRh	1 (4%)	1 (7%)
No response	15 (58%)	9 (60%)

Abbreviations: ALL, acute lymphoblastic leukaemia; allo‐HCT, allogeneic haematopoietic cell transplant (allo‐HSCT); AML, acute myeloid leukaemia; CR, complete remission; CRh, complete remission with partial haematological recovery; CRi, complete remission with incomplete count recovery; ELN, European LeukemiaNet; MDS/AML, myelodysplastic syndrome/acute myeloid leukaemia; MPAL, mixed phenotype acute leukaemia.

^a^ ELN 2022 risk classification only applied to patients with AML (*n* = 19).

Fifteen patients (58%) received revumenib for *KMT2A*r, 10 (38%) for an *NPM1*m and 1 (4%) for a *KMT2A* partial tandem duplication (PTD) (Figure S1A,B). The patient with *KMT2A*‐PTD previously received all available standard therapies and was therefore treated with revumenib given preclinical rationale for menin inhibition.^10^ Twenty patients had a comprehensive molecular evaluation at initial diagnosis. Aside from *KMT2A*r and *NPM1*m, common pathogenic mutations at diagnosis included fms‐related tyrosine kinase 3‐internal tandem duplication (*FLT3‐ITD*) (5/20, 25%), neuroblastoma RAS viral oncogene homolog (*NRAS*) (5/20, 25%), Kirsten rat sarcoma viral oncogene homolog (*KRAS*) (4/20, 20%), tet methylcytosine dioxygenase 2 (*TET2*) (4/20, 20%) and tumor Protein P53 (*TP53*) (2/20, 10%) (Figure S1A).

Responses to revumenib are summarized in Table 1; the ORR rate was 42% (11/26). Fifteen patients received additional treatment after revumenib (post‐revumenib treatment). Of the 11 patients who did not receive post‐revumenib treatment, 10 (91%) died either while taking revumenib, after cessation due to no response, or due to other complications of their leukaemia, while 1 (9%) remained on revumenib at the time of data cut‐off (Figure 1B). The median OS of the 26‐patient cohort from the time of revumenib initiation was 7.3 months (95% confidence interval (CI) [2.5, 14.4]). Fourteen patients had repeat molecular assessments via next‐generation sequencing (NGS) at the time of no response or progression on revumenib (Figure S1C), of which 5 (36%) developed a multiple endocrine neoplasia 1 (*MEN1*) mutation, a known driver of resistance to revumenib.^11^ Of the 5 patients who developed *MEN1* mutations, 3 (60%) had *NPM1*m and 2 (40%) had *KMT2A*r.

**FIGURE 1:**
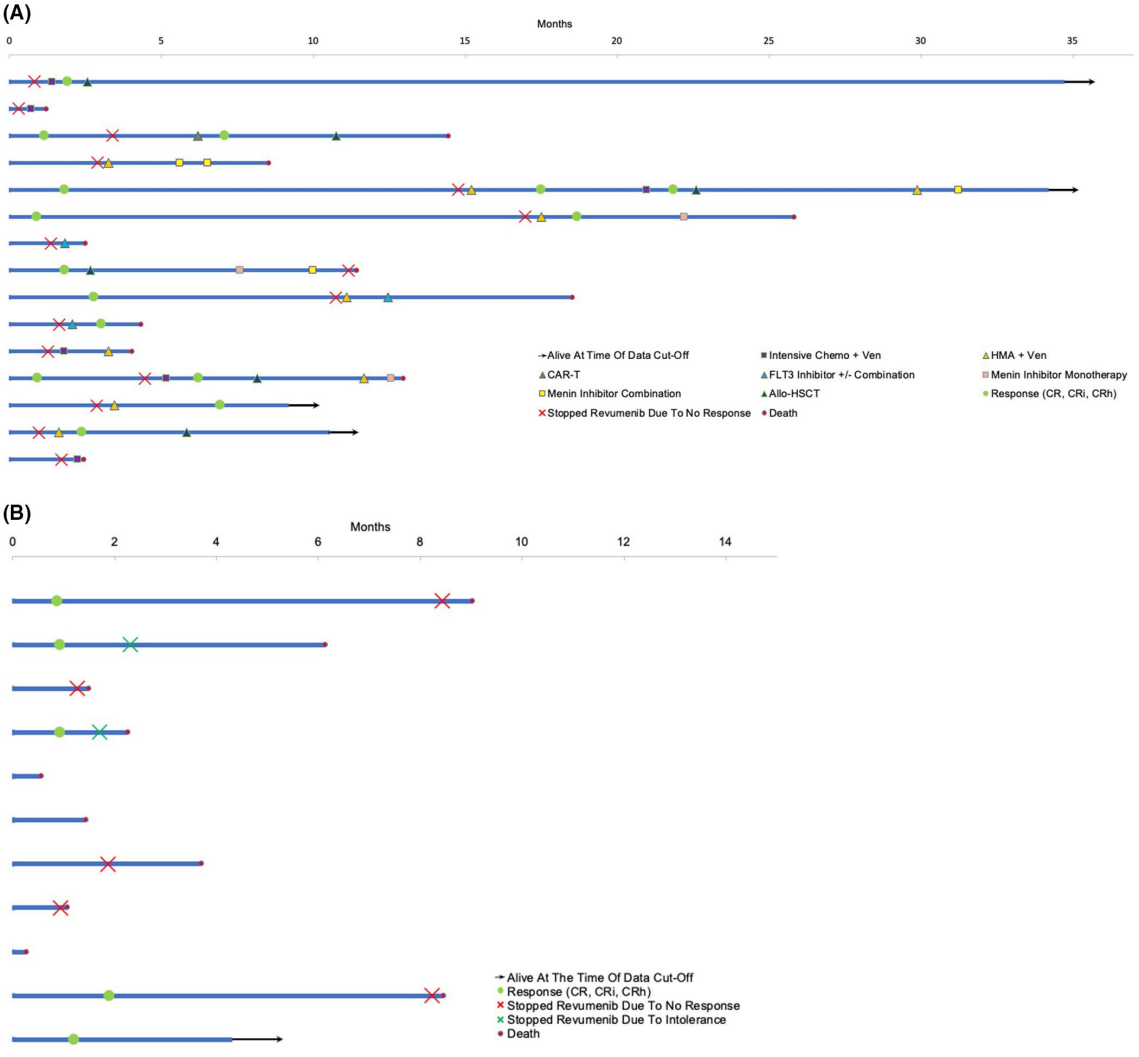
Swimmer plots of patients from revumenib initiation. (A) 15 patients who received additional therapies after revumenib. (B) 11 patients who did not receive additional therapy after revumenib. allo‐HSCT, allogeneic haematopoietic cell transplant (allo‐HCT); CAR‐T, Chimeric Antigen Receptor T‐cell therapy; CR, complete remission; CRh, complete remission with partial haematological recovery; CRi, complete remission with incomplete count recovery; HMA, hypomethylating agent; Ven, venetoclax.

Of the 15 patients who received post‐revumenib treatment, 5 (33%) had an *NPM1* mutation, 9 (60%) had *KMT2A*r and 1 (7%) had a *KMT2A*‐PTD. Twelve patients (80%) received one or two lines of treatment following revumenib, and three patients (20%) received ≥3 lines of treatment (Table S1). As the first line of post‐revumenib treatment, 6 (40%) received a hypomethylating agent (HMA) + venetoclax (ven), 5 (33%) received intensive chemotherapy (IC) + ven, 2 (13%) with *FLT3* mutations received gilteritinib‐based therapy, 1 (7%) with B‐ALL received CD19‐directed chimeric antigen receptor T‐cell (CAR T) therapy and 1 (7%) received revumenib again after previously achieving a CR on revumenib, undergoing allogeneic haematopoietic stem cell transplant (allo‐HSCT), and having another relapse of disease.

Of the 26 patients, 18 (69%) received ven prior to revumenib. Of these, five (28%) achieved a response to revumenib. Nine patients (50%) went on to receive post‐revumenib therapy. Seven of the nine patients (78%) were retreated with ven‐containing regimens post‐revumenib. Two of seven patients (29%) achieved a response to a ven‐containing regimen post‐revumenib.

Following the first line of post‐revumenib treatment, 8 patients (53%) had no response. Of these, 5 (62.5%) had *KMT2A*r, 2 (25%) had *NPM1* mutations and 1 (12.5%) had a *KMT2A‐*PTD. Three patients achieved a CR while four achieved a CRi for an ORR of 47% (Table S1). Among these 7 patients who achieved a response, 3 (42.8%) received HMA + ven, 2 (28.8%) received IC + ven, 1 (14%) received gilteritinib‐based therapy and 1 (14%) received CAR‐T therapy. Among the seven patients with a response after the first line of therapy post‐revumenib, three (43%) had *KMT2A*r and four (57%) had an *NPM1* mutation. Among the four patients with an *NPM1* mutation who achieved a response after their first line of post‐revumenib therapy, *NPM1* measurable residual disease (MRD) status was assessed via NGS assay with 10^−5^ sensitivity. Two patients (50%) were MRD positive at assessment post‐revumenib, and 2 (50%) achieved and maintained MRD‐negative status. Of the two patients with MRD‐positive disease, one patient switched to HMA therapy and has not had subsequent *NPM1* MRD assessments at the time of data cut‐off, and the other patient died prior to repeat MRD assessment. Four patients who received post‐revumenib therapy had a *MEN1* mutation. Two of these patients had *NPM1*m disease and received HMA + ven; both patients achieved a response. Two patients had *KMT2A*r and received HMA + ven but did not have a response.

Among the 15 patients who received post‐revumenib treatment, the median OS was 7.5 months (95% CI [2.3, NA]) from the time of first post‐revumenib treatment and was 8.3 months (95% CI [7.8, NA]) in the seven patients who achieved a CR/CRi to their first post‐revumenib treatment (Figure 1A).

Of the 15 patients, 7 (47%) received additional treatment lines beyond the first line of post‐revumenib therapy; their courses are summarized in the swimmer plots (Figure 1A). Of the post‐revumenib treatment cohort, four patients (27%) were still alive at the time of data cut‐off (Figure 1A). Of the 15 patients who received post‐revumenib therapy, 6 (40%) subsequently underwent allo‐HSCT; three patients died from relapsed disease and three are still alive at the time of data cut‐off. The median OS from the time of allo‐HSCT was 8.8 months (95% CI [4.8, NA]).

In summary, this analysis characterized the clinical outcomes of 26 patients with R/R acute leukaemia treated with revumenib. The ORR was 47% in the 15 patients who received post‐revumenib therapy, with most patients receiving ven‐containing regimens (*n* = 11) as the next line of treatment. Responses were seen in patients with *KMT2A* aberrations (3/10), *NPM1* mutations (4/5) and *MEN1* mutations (2/4), suggesting that additional therapy may be effective across the common aberrations seen after relapse/progression on revumenib. Of the 15 patients who received post‐revumenib therapy, 6 were able to proceed to an allo‐HSCT. Prior work has demonstrated that responses to menin inhibitors in the R/R setting are typically limited in duration and that if MRD‐negative status can be achieved, patients benefit from proceeding to transplant promptly.^5^, ^6^, ^12^

Limitations of our analysis include the small sample size. Many patients received revumenib through a clinical trial or through an expanded access programme. Therefore, our findings may not reflect the broader population which has more limited access to such resources. Nevertheless, our study provides insight into potential therapeutic strategies for revumenib‐exposed patients.

To our knowledge, this is the first report describing outcomes of subsequent therapies after revumenib exposure in a real‐world cohort. We found that post‐revumenib therapies, particularly venetoclax‐based approaches, can induce responses across mutational subsets including *MEN1*. In addition, consolidation with allo‐HSCT is feasible in eligible patients who achieve a response. Prospective studies evaluating therapies, including other menin inhibitors, in patients already treated with revumenib will be critical to understand how to improve outcomes in this group.^13^ As menin inhibitors are investigated in the front‐line setting, similar studies might inform combinatorial trial designs and identify effective treatments after menin inhibitor therapy.^14^, ^15^

## AUTHOR CONTRIBUTIONS

Miles Thomas was responsible for project design, data collection, data analysis and manuscript creation. Hannah Johnston was responsible for project design, data collection, data analysis and manuscript creation. Emily Dworkin was responsible for manuscript creation. Austin Wesevich was responsible for manuscript creation. Gregory W. Roloff was responsible for manuscript creation. Caner Saygin was responsible for manuscript creation. Mariam T. Nawas was responsible for manuscript creation. Michael W. Drazer was responsible for manuscript creation. Adam S. DuVall was responsible for manuscript creation. Satyajit Kosuri was responsible for manuscript creation. Michael J. Thirman was responsible for manuscript creation. Olatoyosi Odenike was responsible for manuscript creation. Wendy Stock was responsible for manuscript creation. Richard A. Larson was responsible for manuscript creation. Rafael Madero‐Marroquin was responsible for project concept and design, data collection, data analysis and manuscript creation. Anand A. Patel was responsible for project concept and design, data collection, data analysis and manuscript creation.

## FUNDING INFORMATION

Anand A. Patel is supported by the NCI Early Career Investigator Award (3P30CA014599‐49S1).

## CONFLICT OF INTEREST STATEMENT

Miles Thomas: No conflicts of interest to disclose. Hannah Johnston: No conflicts of interest to disclose. Emily Dworkin: Honoraria from AbbVie. Austin Wesevich: Honorarium from Amgen. Gregory W. Roloff: Advisory boards for Autolus Therapeutics and Kite/Gilead. Caner Saygin: No conflicts of interest to disclose. Mariam T. Nawas: No conflicts of interest to disclose. Michael W. Drazer: Scientific advisory board for Argenx. Adam S. DuVall: Speaker for CE Concepts. Satyajit Kosuri: No conflict of interest to disclose. Michael J. Thirman: Has acted as a consultant or advisor to AbbVie, AstraZeneca, Celgene, Janssen, Pharmacyclics and Roche/Genentech. Research funding from AbbVie (Inst), Gilead Sciences, Janssen, Merck, Nurix, Pharmacyclics, Syndax and TG Therapeutics. Olatoyosi Odenike: Institutional research funding by AbbVie, Astra Zeneca, Celgene, Curis, Incyte, Shattuck Lab and K‐group alpha; scientific advisory board participant for AbbVie, Celgene/BMS, Novartis, Incyte, Kymera therapeutics, Servier and Rigel; service on data safety board for Treadwell therapeutics. Wendy Stock: Advisor for Kura, Servier, Newave and Asofarma. Richard A. Larson has acted as a consultant or advisor to Ariad/Takeda, CVS/Caremark, Epizyme/Ipssen and Novartis and has received clinical research support to his institution from Astellas, Biomea, Cellectis, Daiichi Sankyo, Forty Seven/Gilead and Novartis and royalties from UpToDate. Rafael Madero‐Marroquin: No conflicts of interest to disclose. Anand A. Patel: Honoraria from AbbVie, Amgen, Astellas, Jazz, Sobi, Syndax; research funding (institutional) from Pfizer, Incyte, Servier and Sumitomo.

## PATIENT CONSENT STATEMENT

Informed consent was waived as per institutional review board approval due to the retrospective nature of the study.

## PERMISSION TO REPRODUCE MATERIAL

Those seeking to reproduce material from this manuscript should reach out to the corresponding author for permission.

## Supporting information


Figure S1.

Table S1.

## Data Availability

The data used for this study can be made available in its de‐identified form at the request of the corresponding author upon reasonable request and following institutional data sharing practices. To protect the confidentiality and security of individual health records, these data will not be made publicly available.
