# Supplementary figures and images for: Outcomes of patients with relapsed/refractory acute leukaemia treated with revumenib with a focus on post‐revumenib therapies
Source: Br J Haematol. 2025 Oct 26;208(1):343–7. doi: 10.1111/bjh.70225 (PMC12819088; doi:10.1111/bjh.70225)

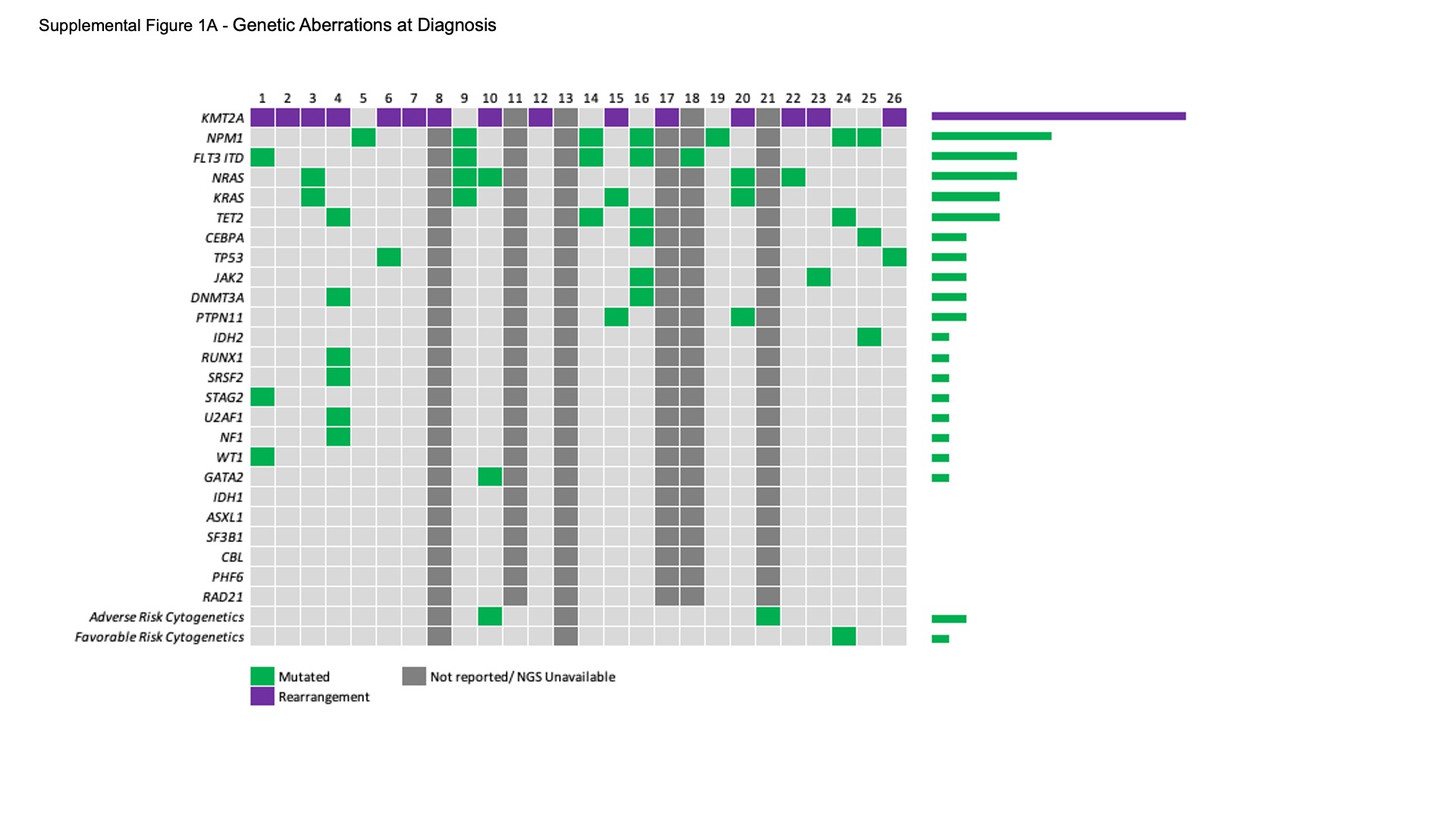

Supplement: Supplementary file 1 — Figure S1. [file BJH-208-343-s001.zip › bjh70225-sup-0001-FigureS1@Revisions_Supplemental Figure 1A .jpg]

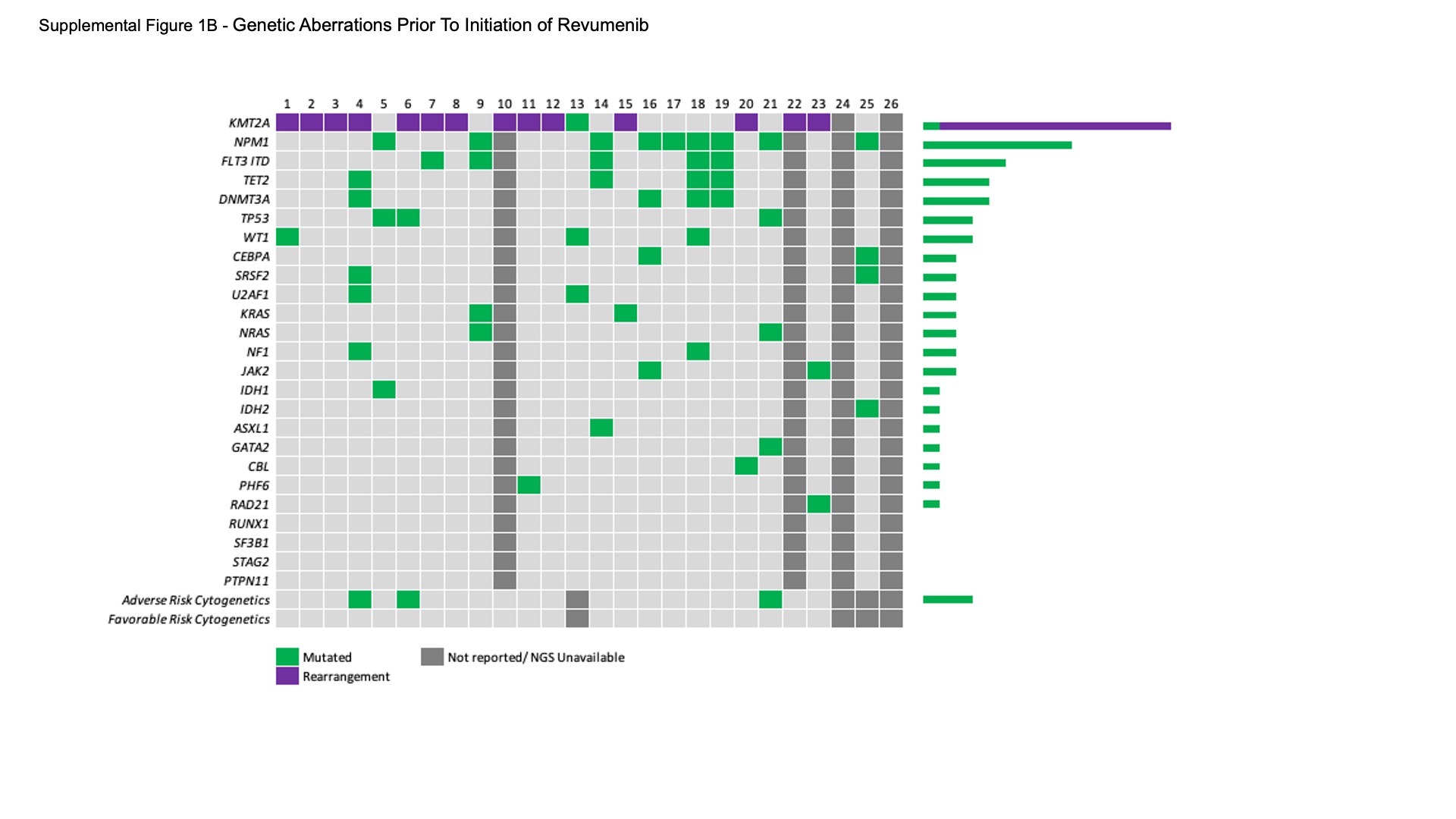

Supplement: Supplementary file 1 — Figure S1. [file BJH-208-343-s001.zip › bjh70225-sup-0002-FigureS1@Revisions_Supplemental Figure 1B.jpg]

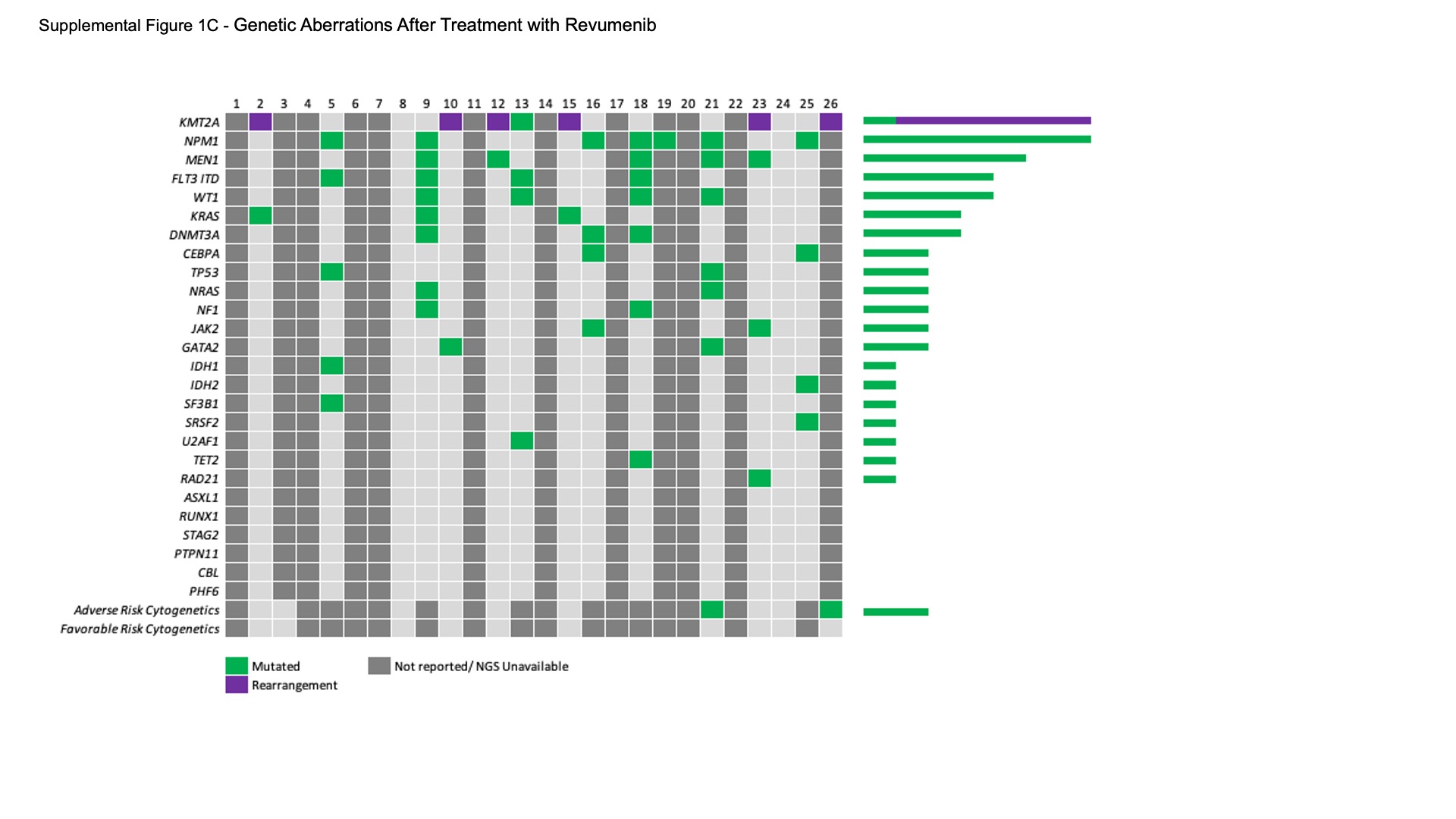

Supplement: Supplementary file 1 — Figure S1. [file BJH-208-343-s001.zip › bjh70225-sup-0003-FigureS1@Revisions_Supplemental Figure 1C.jpg]
