# Supplementary material for: Outcomes of patients with relapsed/refractory acute leukaemia treated with revumenib with a focus on post‐revumenib therapies
Source: Br J Haematol. 2025 Oct 26;208(1):343–7. doi: 10.1111/bjh.70225 (PMC12819088; doi:10.1111/bjh.70225)
Supplement: Supplementary file 2 — Table S1. [file BJH-208-343-s002.docx]

**Supplementary Table 1: Treatment Characteristics and Clinical Outcomes for Patients Who Received Post-Revumenib Therapy**

|  | **Patients who received post-revumenib treatment, n = 15 (%)** |
| --- | --- |
| **Lines of treatment post-revumenib** | |
| 1 | 7 (47%) |
| 2 | 5 (33%) |
| ≥ 3 | 3 (20%) |
| **Allo-HSCT post-revumenib** | 6 (40%) |
| **Response to first post-revumenib line of treatment** | |
| CR | 3 (20%) |
| CRi | 4 (27%) |
| CRh | 0 (0%) |
| No Response | 8 (53%) |
| **ORR* by ELN 2022 risk classification at diagnosis, number of responses/total (%)**** | |
| Favorable | 3/3 (100%) |
| Intermediate | 0/4 (0%) |
| Adverse | 2/4 (50%) |
| **ORR by first line of post-revumenib line therapy, number of responses/total (%)** | |
| Intensive chemo + venetoclax | 2/5 (40%) |
| HMA + venetoclax | 3/6 (50%) |
| Gilteritinib +/- additional therapy | 1/2 (50%) |
| CAR T | 1/1 (100%) |
| Revumenib monotherapy | 0/1 (0%) |

*Allo-HSCT = allogeneic hematopoietic cell transplant; AML = acute myeloid leukemia; CAR-T = Chimeric Antigen Receptor T cell therapy; CR = Complete Remission; CRh = Complete Remission with Partial Hematologic Recovery (CRh); CRi = Complete Remission with Incomplete Count Recovery; ELN = European LeukemiaNet; HMA = hypomethylating agent; ORR = overall response rate.*

**ORR was defined as CR+CRi+CRh*

***Response rates by ELN risk stratification were evaluated for patients with AML only (n=11)*
